# Supplementary material for: The Role of Social Media in Improving Patient Recruitment for Research Studies on Persistent Post-Infectious Olfactory Dysfunction
Source: Medicina (Kaunas). 2022 Feb 25;58(3):348. doi: 10.3390/medicina58030348 (PMC8949697; doi:10.3390/medicina58030348)
Supplement: Supplementary file 1 [file medicina-58-00348-s001.zip › medicina-1589256-supplementary.pdf]

ON-LINE SURVEY

Title of the study: The role of social media in improving patient recruitment for research studies on persistent post-infectious olfactory dysfunction

1. What is your age?
   _ _ years

2. What is your sex?
   - Male
   - Female

3. When did you lose your sense of smell?

   _ _ / _ _ / _ _ _ _

4. Was that due to SARS-CoV-2 (COVID-19)?
   - Yes (Tested positive)
   - No (Tested negative)
   - Not sure (Never tested)
   - Other viral infection

5. How was your sense of smell when you lost it? Please rate on a scale from 0 (Absent) to 10 (Normal – not affected)

   _ _ out of 10

6. How is your sense of smell today? Please rate on a scale from 0 (Absent) to 10 (Normal – as it was before the infection)

   _ _ out of 10

7. Do you have parosmia (distorted sense of smell)?
   - Yes
   - No

8. Do you have phantosmia (smelling things that have no obvious origin)?
   - Yes
   - No

9. Is your sense of smell improving?
   - Yes
   - No
   - Not sure

10. How long have you been doing olfactory training?
    _ _ (months)

11. Have you found an improvement whilst doing olfactory training?
    - Yes
    - No
    - Not sure

12. Have you tried other medications in the past to improve your sense of smell? Please indicate in the box below.

    ____

13. Have any of these other medications helped you?
    - Yes. Please indicate if there is a specific treatment from which you benefitted more.

    ____
    - No
    - Not sure

14. Would you be willing to take part in a study looking at new treatment options to improve sense of smell after post-viral infections?
    - Yes
    - No

17 If yes, what kind of study would you be interested in?
    - Medical (new medication)
    - Surgical (new operation) under general anaesthesia (sleeping)

18 What is your feeling about the possibility of being put into a study in which you do not know what treatment you are going to have? (All patients will receive a surgical treatment)
    - Yes, I will be interested
    - No, I will not be interested
